# Diabetic Retinopathy and Regulation of Mitochondrial Glutathione–Glutathione Peroxidase Axis in Hyperhomocysteinemia

**DOI:** 10.3390/antiox13030254

**Published:** 2024-02-20

**Authors:** Pooja Malaviya, Renu A. Kowluru

**Affiliations:** 1 Ophthalmology, Visual and Anatomical Sciences, Wayne State University, Detroit, MI 48202, USA; 2 Kresge Eye Institute, Wayne State University, Detroit, MI 48201, USA

**Keywords:** diabetic retinopathy, DNA methylation, glutathione, peroxidase, homocysteine, mitochondria

## Abstract

Diabetic patients have elevated homocysteine levels, and hyperhomocysteinemia is shown to exacerbate mitochondrial damage, which plays a central role in diabetic retinopathy. Glutathione peroxidases (GPx) catalyze hydrogen peroxide (H_2_O_2_) reduction using glutathione (GSH) as a cofactor. GSH and GPx are mainly cytosolic but are also present in the mitochondria to neutralize H_2_O_2_ produced by superoxide dismutase, and in diabetes, they are downregulated. Hyperhomocysteinemia also disrupts the balance between S-adenosyl-L-homocysteine and S-adenosylmethionine (SAM); SAM is also a methyl donor for DNA methylation. The aim of this study was to investigate the role of homocysteine in mitochondrial GSH–GPx1 regulation in diabetic retinopathy. Human retinal endothelial cells in 20 mM D-glucose + high homocysteine were analyzed for ROS, GSH and GPx in the mitochondria, and SAM levels and *GPx1* promoter DNA methylation were also studied (5-methylcytosine and MS-PCR). The results were confirmed in the retina from streptozotocin-induced hyperhomocysteinemic (cystathionine-β-synthase-deficient) diabetic mice. High homocysteine exacerbated the glucose-induced decrease in GSH levels and GPx activity in the mitochondria and the downregulation of *GPx1* transcripts and further increased SAM levels and *GPx1* promoter DNA methylation. Similar results were obtained in a hyperglycemic–hyperhomocysteinemic mouse model. Thus, elevated homocysteine in diabetes hypermethylates *GPx1* promoter, thus decreasing the mitochondrial GPx/GSH pool and exacerbating mitochondrial damage. Modulating hyperhomocysteinemia could be a potential therapeutic avenue to target mitochondrial dysfunction in diabetic retinopathy.

## 1. Introduction

Retinopathy is one of the most common complications of diabetes; although classified as a microvascular disease, neurodegeneration also contributes to its pathogenesis. Many metabolic, molecular, functional and structural abnormalities have been implicated in its development, but the exact molecular mechanism of this progressive disease remains unclear [1,2]. Oxidative stress, an imbalance between the formation and removal of free radicals, is considered to play a critical role in the development of diabetic retinopathy [3,4,5]. In diabetes, the retina experiences a double whammy, while the production of free radicals is increased, and the antioxidant defense system, including intracellular antioxidant glutathione (GSH) and the antioxidant defense enzymes superoxide dismutase and glutathione peroxidase (GPx), are downregulated [6,7].

Mitochondria, in addition to being the powerhouse of a cell, are also a major source of free radicals, and their homeostasis, which involves an array of processes, including structural, functional and genomic stability, is critical for cell survival [4,8]. In the pathogenesis of diabetic retinopathy, retinal mitochondrial homeostasis is impaired, free radical production is increased and the electron transport chain system is compromised, fueling into a self-propagating vicious cycle of free radicals [3,4]. Mitochondria are also equipped with a good antioxidant defense system, including a superoxide scavenging enzyme, manganese superoxide dismutase [9]. Although GSH is synthesized in the cytosol, mitochondria contain 10–15% of the total cellular GSH (mt-GSH). In addition, selenium-dependent glutathione peroxidases, especially GPx1 and GPx4, are also located in the mitochondria, where they detoxify H_2_O_2_ generated from the dismutation of superoxide and lipid peroxides, respectively, using GSH as a cofactor [10,11,12], and a decrease in GPx4-GSH is intimately associated with ferroptosis, an iron-dependent cell death seen in diabetic retinopathy [13,14].

Hyperglycemia is the main instigator in the development of diabetic retinopathy, but many systemic factors also contribute to its development [15], and homocysteine, a sulfur-containing non-protein amino acid, is one of them [16]. Diabetic patients have increased levels of circulating homocysteine, and visual function and the blood–retinal barrier are impaired in animals genetically manipulated to accumulate increased homocysteine levels [16,17,18,19]. Moreover, high homocysteine accelerates and exacerbates mitochondrial damage and the development of diabetic retinopathy in animal models [19,20]. Homocysteine is biosynthesized by the methionine–homocysteine metabolism in a process involving methyl group donation by S-adenosylmethionine (SAM), which in turn is converted into S-adenosylhomocysteine, a precursor of homocysteine. Elevated levels of homocysteine disrupt a delicate balance between methyl donor SAM and S-adenosylhomocysteine. Homocysteine can either be remethylated back to L-methionine or, via trans-sulfuration, to L-cysteine, and cysteine is an important amino acid for the biosynthesis of GSH [21,22,23]. How hyperhomocysteinemia affects the GPx–GSH balance in diabetic retinopathy is unclear.

Gene expression can also be regulated by epigenetic modifications without altering the DNA sequence [24], and DNA methylation machinery is activated in diabetes, resulting in the aberrant expression of many retinal genes implicated in the pathogenesis of diabetic retinopathy [4]. The promoter region of *GPx1* has GC-boxes for Sp1 family transcription factors, and aberrant hypermethylation of *GPx1* promoter DNA is implicated in its downregulation in gastric cancer [25,26]. S-adenosylmethionine is the main methyl donor used by DNA methyltransferases (Dnmts) for DNA methylation [27], but the role of hyperhomocystemenia in *GPx1* promoter DNA methylation in diabetic retinopathy is not elucidated. Our aim was to investigate the role of homocysteine in the regulation of mitochondrial GSH–GPx1 in diabetic retinopathy. 

## 2. Methods

Human retinal endothelial cells: Primary human retinal endothelial cells (HRECs, Cat. No. ACBRI 181, Cell Systems Corp., Kirkland, WA, USA) were cultured in Dulbecco’s modified Eagle medium (DMEM, Cat. No. D5523, Sigma-Aldrich, St. Louis, MO, USA) supplemented with 12% fetal bovine serum, 20 μg/mL endothelial cell growth supplement and 1% each of glutamax, insulin–transferrin–selenium and antibiotic/antimycotic at 37 °C in a 5% CO_2_ environment. Cells (80–90% confluent) from the 6th–8th passage were incubated for 96 h in a culture medium (1% fetal calf serum, 9% Nu-serum and 1 μg/mL endothelial growth supplement) supplemented with either 5 mM D-glucose (NG) or 20 mM D-glucose (HG) in the presence or absence of 100 µM L-homocysteine thiolactone hydrochloride (Cat. No. S784036, Sigma-Aldrich; Hcy group). Each experiment also included a parallel incubation of HRECs in 20 mM L-glucose (L-Gl), which served as an osmotic/metabolic control [20,23]. Our previous study has shown that hyperhomocysteinemia alone (without hyperglycemia) does not cause significant mitochondrial damage [22]; a group containing cells in 5 mM glucose + 100 µM L-homocysteine was not included in the present study. 

A group of cells from the 5th–7th passage were transfected with either silencing the enzyme implicated in homocysteine metabolism, cystathionine β-synthase (*Cbs*), employing the siRNA of *Cbs* (Cat. No. 4392421, siRNA ID: s528455, Thermofisher Scientific, Waltham, MA, USA) or of methionine adenosyltransferase 1a (*Mat1a*) (Cat. No. 4392420, siRNA ID: s8524, Thermofisher Scientific), using Lipofectamine™ RNAiMAX Transfection Reagent (Cat. No. 13778150, Invitrogen™, Carlsbad, CA, USA), as described previously [28]. As a control, each transfection experiment had cells transfected with a non-targeting scrambled control RNA (Cat. No. AM4611, Thermofisher Scientific). After 8 h of incubation with the siRNA, the cells were washed with DMEM and incubated for 96 h in a medium containing 20 mM D-glucose.

Mice: A colony of mice, encompassing *Cbs*^+*/*+^ and *Cbs*^+*/−*^, was established using breeding pairs of *Cbs*^+/−^ mice (B6.129P2-Cbstm1Unc/J, Jackson Laboratories, Bar Harbor, ME, USA) [29]. Diabetes was induced in 8–10-week-old mice (male and female) by intraperitoneal injection of 55 mg/kg streptozotocin for three consecutive days. Mice were maintained as diabetic (blood glucose > 350 mg/dL) for ~4 months. Each diabetic group had similar numbers of male and female mice. The control group included age- and sex-matched nondiabetic *Cbs*^+*/*+^ mice. At the end of the experiment, the retina was isolated for its biochemical parameters.

Gene transcripts: TRIZOL-extracted RNA (1 µg) was processed for cDNA synthesis using a High-Capacity cDNA Reverse Transcription kit (Cat. No. 4368814, Applied Biosystems, Waltham, MA, USA) and was employed to perform quantitative real-time PCR (qRT-PCR) employing gene- and species-specific primers (Table 1) and SYBR green (Cat. No. 4367659, Applied Biosystems). The relative fold change in the gene expression was calculated using the delta–delta Ct method and *β-actin* (HRECs) or *18s rRNA* (mouse) as the housekeeping gene [7].

Homocysteine: Using 15 µg of protein, total homocysteine was measured by an ELISA method (Cat. No. MBS2883009, MyBioSource, San Diego, CA, USA), and the final absorbance was measured at 450 nm, as reported previously [7].

Subcellular fractionation: Cells/retina were homogenized in 30 mM Tris-HCl buffer (pH 7.5) containing 2 mM EGTA, 1 mM EDTA, 1% Triton X-100, 250 mM sucrose, 1 mM sodium fluoride, 1 mM phenylmethylsulfonyl fluoride, 1 mM sodium orthovanadate and 1 μg/mL protease inhibitor mixture, and the sample was centrifuged at 750× *g* for five minutes to remove the cell debris. The resultant supernatant was used for analyzing the total GPx and GSH levels. 

Mitochondria were prepared by homogenizing cells/retina in the mitochondrial buffer (25 mM Tris-HCl, pH 7.4, 250 mM sucrose, 2 mM EDTA and 1 μg/mL protease inhibitor mixture) using a glass homogenizer. The homogenate was centrifuged at 750× *g* for five minutes to remove the cell debris, and the supernatant was then centrifuged at 10,000× *g* for 15 min. The resulting mitochondrial pellet was washed with the mitochondrial buffer by centrifuging at 10,000× *g* for 15 min and then suspended in the mitochondria buffer [30].

Nuclear proteins were extracted using an EpiQuick Nuclear Extraction kit (Cat. No. OP-0002, EPIGENTEK, Farmingdale, NY, USA) following manufacturer’s protocol. Briefly, the cell pellet was washed with PBS and centrifuged at 750× *g* for five minutes and then suspended in pre-extraction buffer (NE1) containing dithiothreitol and protease inhibitors. For the retina, small pieces were homogenized in NE1. The suspension (cell or retina) was incubated on ice for 10 min, vortexed for 10 s and centrifuged at 11,000× *g* for 1 min. The nuclear pellet was suspended in NE1 buffer containing dithiothreitol and protease inhibitors (1:1000 each) and centrifuged at 11,000× *g* for one minute. The final nuclear pellet was suspended in the extraction buffer, incubated on ice for 15 min and sonicated for 3 × 10 s. The suspension was then centrifuged at 12,500× *g* for 10 min, and the supernatant was used for quantifying the enzyme activity of Dnmt [31].

Glutathione: GSH levels were quantified by an enzymatic recycling method using a GSH assay kit (Cat. No. 703002, Cayman Chemicals, Ann Arbor, MI, USA). Protein (25 μg, homogenate or isolated mitochondria) was deproteinized by phosphoric acid, and GSH was measured in the supernatant after neutralizing its pH with triethanolamine. The absorbance of 2-nitro-5-thiobenzoate, produced by the reduction of 5,5′-dithio-bis-2 nitrobenzoic acid by GSH, was measured at 412 nm [32].

Reactive oxygen species: Total ROS were quantified fluorometrically; 5 µg protein was incubated with 4µM 2′,7′-dichlorofluorescein diacetate (DCFH-DA, Cat. No. D6883, Sigma-Aldrich), and mitochondrial ROS (mt-ROS) were quantified by incubating 10 µg of mitochondrial protein with 5 µM MitoSox red (Cat. No. M36008, Thermo Fisher Scientific), as reported previously [7]. Cells incubated with 2 μM antimycin A in a 5 mM D-glucose medium for one hour were used as a positive control for mt-ROS.

Glutathione peroxidase activity: A standard glutathione-dependent peroxidase assay was carried out in a 100 μL reaction mixture containing 15 μg of protein (homogenate or mitochondria), as per the manufacturer’s instruction (Cat. No. 703102, Cayman Chemicals). The reaction was initiated by adding cumene hydroperoxide, and the reduction in absorbance at 340 nm was monitored spectrophotometrically for 5 min. The initial rate of reaction was calculated using the linear portion of the curve, and GPx activity was expressed as the amount of NADPH oxidized per minute per µg of protein [33].

S-adenosyl methionine: Cell/tissue lysate was prepared by sonication in ice-cold PBS using a 30 kHz sonicator with probe at 30% amplitude for three cycles of 10 s each. SAM was quantified in the cell-free supernatant using an S-adenosylmethionine ELISA kit from Cell Biolabs, INC. (Cat. No. MET-5152, Cell Biolabs, San Diego, CA, USA), following the manufacturers’ protocol. Final absorbance was measured at 450 nm using an ELISA plate reader [34].

Activity of Dnmts: The enzyme activity of Dnmts was quantified in the nuclear fraction (5 μg of protein) by an EpiQuik™ DNA Methyltransferase Activity assay kit (Cat. No. P-3001, EPIGENTEK) by quantifying methylated DNA using a 5-methylcytosine (5mC) antibody, as described previously [23].

DNA methylation: The presence of CpG islands in the *GPx1* promoter was identified by MethPrimer [35], and 5mC levels were quantified by a methylated DNA immunoprecipitation (MeDIP) method. Briefly, genomic DNA, isolated using a DNeasy kit (Cat. No. 69504, Qiagen, Valencia, CA, USA), was incubated with 5mC antibodies to immunoprecipitate 5mC-enriched DNA (MeDIP kit, Cat. No. P-1015, EPIGENTEK, Farmingdale, NY, USA), and 5mC at the *GPx1* promoter was quantified by qRT-PCR [23].

DNA methylation of *GPx1* promoter was confirmed by methylation-specific PCR (MS-PCR). DNA (2 µg), sheared into single-stranded DNA, was subjected to bisulfite treatment using an EpiTect Bisulfite kit (Cat. No. 59104, Qiagen), and MS-PCR was performed using methylation- and unmethylation-specific primers (Table 1). PCR products were analyzed on a 2% agarose gel, and the ratio of the band intensities of methylated DNA to unmethylated DNA was quantified [36].

*GPx1* promoter DNA methylation was confirmed by two independent methods, the MeDIP and MS-PCR techniques; while the MeDIP method quantitatively measures 5mC levels, the MS-PCR method is qualitative, which selectively amplifies DNA based on its methylation status, and the use of primers designed specifically for either methylated or unmethylated DNA sequences allows for the selective amplification of the target region based on its methylation status. Compared to the cumbersome and costly bisulfite Sanger sequencing with its relatively low read counts, the MS-PCR method is a cost-effective technique that can be carried out in a few hours and is sensitive in detecting low levels of methylated DNA. Although bisulfite next-generation sequencing is more accurate than MS-PCR, this very costly technique requires more sample and time for sample preparation, sequencing and data analysis and validation by pyrosequencing or MS-PCR [37,38].

Statistical analysis: Statistical analysis was carried out using Graphpad Prism (version 8). Data are presented as mean ± SD of three or more experiments, each performed in triplicate. Comparison between groups were made using one-way ANOVA followed by Student’s *t*-test, and a *p* value less than 0.05 was considered statistically significant.

## 3. Results

### 3.1. In Vitro Human Retinal Endothelial Cells

**Figure 1:**
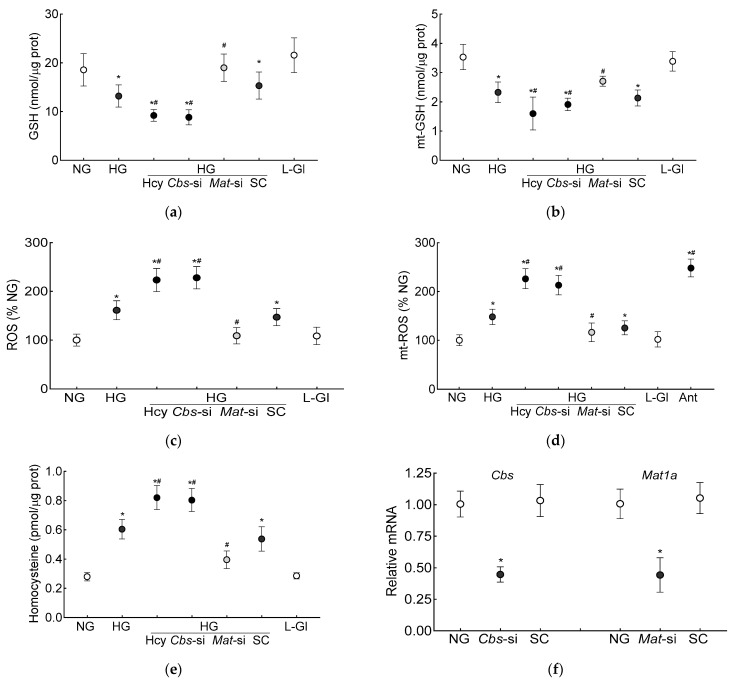
Effect of homocysteine on high glucose-mediated mitochondrial GSH-ROS in human retinal endothelial cells. Cells incubated in high-glucose + high-homocysteine conditions (100 µM L-homocysteine thiolactone hydrochloride or transfected with *Cbs-*siRNA) were analyzed for (**a**) total and (**b**) mitochondrial GSH by quantifying the reduction of 5,5′-dithio-bis-2 nitrobenzoic acid by GSH and (**c**) total ROS levels by DCFH-DA and (**d**) mitochondrial ROS levels by MitoSox red. (**e**) Homocysteine levels were quantified by ELISA. (**f**) The transfection efficiency of *Cbs*-siRNA and *Mat1a*-siRNA was determined by quantifying their respective gene transcripts by qRT PCR using *β-actin* as housekeeping gene. Results are presented as mean ± SD from three different cell preparations, with each measurement made in triplicate. NG and HG = 5 mM and 20 mM D-glucose, respectively; HG/Hcy = 20 mM D-glucose + 100 µM homocysteine; HG/*Cbs-*si and HG/*Mat*-si *=* cells transfected with *Cbs-*siRNA or *Mat1a-*siRNA and incubated in 20 mM D-glucose; HG/SC = scrambled control RNA-transfected cells in 20 mM D-glucose; L-Gl = 20 mM L-glucose; Ant = 5 mM glucose + 2 μM antimycin A. * *p* < 0.05 vs. NG; ^#^ *p* < 0.05 vs. HG.

The GSH levels (total and mitochondrial), as expected [7], were significantly decreased and ROS levels were increased in the high-glucose conditions. Additional homocysteine in the high-glucose conditions, either by supplementing the medium with homocysteine or by silencing *Cbs* (*Cbs*-siRNA), further decreased the total and mt-GSH and increased the total and mt-ROS levels. The values obtained from the cells supplemented with homocysteine or *Cbs*-siRNA-transfected cells in the high-glucose conditions, were not different from each other but were significantly different from the control scrambled RNA-transfected cells in the high-glucose conditions (*p* < 0.05; Figure 1a–d). Incubation of the cells in 20 mM L-glucose, instead of 20 mM D-glucose, had no effect on GSH levels, and the values in 20 mM D-glucose and 20 mM L-glucose were significantly different from each other (*p* < 0.05; Figure 1a–d). Figure 1e is included to show the ~3-fold increase in homocysteine levels in the cells supplemented with homocysteine or those transfected with *Cbs*-siRNA and incubated in high-glucose conditions, and Figure 1f demonstrates the transfection efficiency of *Cbs*-siRNA and *Mat1*-siRNA in the HRECs.

**Figure 2:**
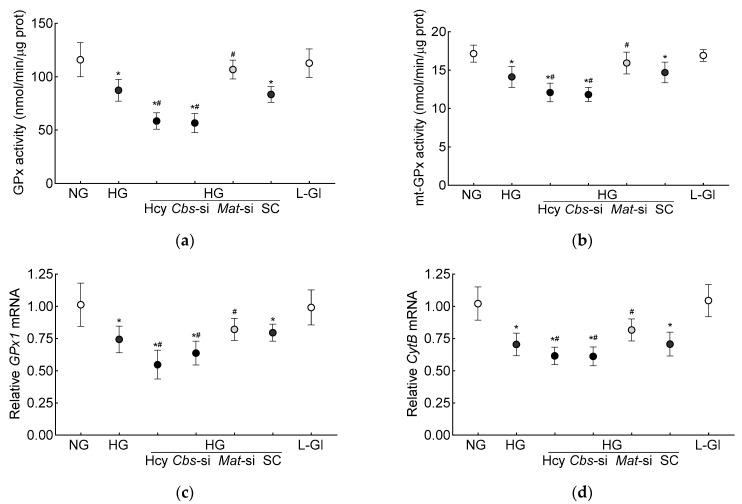
Hyperhomocystenemia and glutathione peroxidase. (**a**) Total and (**b**) mitochondrial GPx activities were measured spectrophotometrically by measuring oxidation of NADPH. Gene transcripts of (**c**) *GPx1* and (**d**) *CytB* were quantified by qRT-PCR using *β-actin* as housekeeping gene. Results are mean ± SD from three or more cell preparations, with each measurement made in triplicate. NG = 5 mM D-glucose; HG and HG/Hcy = 20 mM D-glucose, without or with homocysteine, respectively; HG/*Cbs*-si and HG/*Mat*-si = *Cbs*-siRNA- or *Mat1a*-siRNA-transfected cells in 20 mM D-glucose; HG/SC = scrambled control RNA-transfected cells in 20 mM D-glucose; L-Gl = 20 mM L-glucose; * *p* < 0.05 vs. NG; ^#^ *p* < 0.05 vs. HG.

Glutathione is an important cofactor for GPx1, an intracellular antioxidant enzyme, which reduces hydrogen peroxide to water [10,11]; the effect of hyperhomocysteinemia on GPx activity was determined. Compared to the normal glucose conditions, the GPx activity was inhibited by >30% in the high-glucose conditions, and the inhibition was >50% when both hyperglycemia and homocysteinemia were present (Figure 2a). Consistent with the total GPx activity, the high-glucose conditions also inhibited mt-GPx activity, which was further reduced by the presence of high homocysteine levels (Figure 2b). As with the inhibition of GPx activity, its gene transcripts were also significantly decreased by the high-glucose conditions (*p* < 0.05), and were further decreased by the introduction of homocysteine in the high-glucose medium (*p* < 0.05 vs. HG, Figure 2c). To determine the effect of the decrease in mitochondrial GSH–GPx on mitochondrial damage, transcripts of mtDNA-encoded cytochrome B (*CytB*) of complex III were quantified. As expected [28], *CytB* gene transcripts were decreased by the high-glucose conditions (*p* < 0.05), and the incubation of cells with high homocysteine in the high-glucose medium further decreased *CytB* transcripts (*p* < 0.05 vs. HG, Figure 2d). 

**Figure 3:**
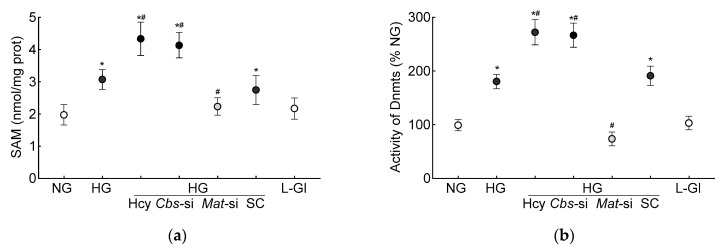

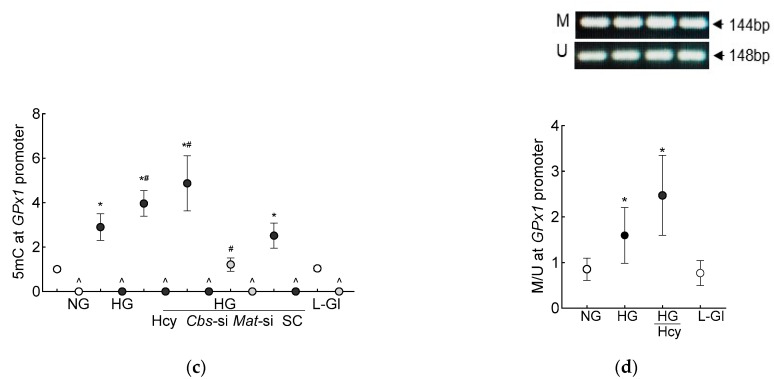
DNA methylation in high homocysteine high glucose conditions. (**a**) SAM levels were quantified by competitive ELISA, (**b**) Dnmts activity was determined by quantifying methylated DNA using an anti-5-methylcytosine antibody, and (**c**) 5mC levels at the *GPx1* promoter in 5mC-enriched DNA were quantified by qRT-PCR. (**d**) Methylation-specific PCR was performed using bisulfite-converted DNA. Results are mean ± SD from 3–4 different cell preparations, with each measurement made at least in duplicate. NG and HG = 5 mM and 20 mM D-glucose, respectively; HG/Hcy = 20 mM D-glucose + 100 µM homocysteine; HG/*Cbs-*si and HG/*Mat*-si *=* cells transfected with *Cbs-*siRNA or *Mat1a-*siRNA and incubated in 20 mM D-glucose; HG/SC = scrambled control RNA-transfected cells in 20 mM D-glucose; L-Gl = 20 mM L-glucose; M = methylated; U = unmethylated. ^ = IgG control; * *p* < 0.05 vs. NG; ^#^ *p* < 0.05 vs. HG.

S-adenosylmethionine, the primary source of methyl groups, is required for transmethylation reactions in homocysteine metabolism, and demethylation of SAM converts to homocysteine via S-adenosylhomocysteine [39]; its levels were quantified in the HRECs. As shown in Figure 3a, the high-glucose conditions increased the SAM levels by 50% and by over two-fold when supplemented with high levels of homocysteine. 

DNA methylation requires the transfer of a methyl group from SAM to the 5′ position of cytosine, and the process is mediated by Dnmts [27]; the effect of homocysteine on the activity of Dnmts was determined. Compared to the normal glucose conditions, the activity of Dnmts was ~2-fold higher in the high-glucose conditions and ~3-fold higher when both high levels of glucose and high levels of homocysteine were present. The values obtained from the cells supplemented with homocysteine or the *Cbs*-siRNA-transfected cells, in the high-glucose conditions, were not different from each other (*p* > 0.05). L-glucose had no effect on the activity of Dnmts (Figure 3b). To investigate the effect of high homocysteine levels on *GPx1* promoter DNA methylation, 5mC levels were quantified. As shown in Figure 3c, glucose increased the 5mC levels at the *GPx1* promoter by about three-fold, but when a high level of homocysteine was also present in the high-glucose medium, the increase in 5mC levels was over four-fold, and the values from the cells in the high-glucose conditions were significantly different from the cells in the high-glucose + high-homocysteine conditions (either by homocysteine supplementation or by *Cbs*-siRNA). The IgG antibody control values in these samples were <1% of the values obtained by the 5mC antibody. Consistent with the 5mC levels, MS-PCR also showed a significant increase in the ratio of methylated bands to unmethylated bands at the *GPx1* promoter in the high-glucose conditions, with or without high levels of homocysteine (*p* < 0.05), and the values from the cells in 5 mM D-glucose or 20 mM L-glucose were not different from each other (Figure 3d). 

The production of SAM from methionine is mediated by Mat1a [39]; to further confirm the role of homocysteine-SAM in mitochondrial GSH–GPx1 downregulation, cells transfected with *Mat1*-siRNA were employed. In the *Mat*-siRNA-transfected cells exposed to a high-glucose milieu, the GSH and ROS levels, total and mitochondrial, were similar to those obtained from the untransfected cells in normal glucose conditions, and the GPx activity was also not different from that obtained from the cells in normal glucose conditions (*p* > 0.05). Consistent with these results, the SAM levels, Dnmts activity and DNA methylation at the promoter of *GPx1* in these *Mat1*-siRNA-transfected cells were significantly different from the untransfected cells in glucose alone or in the glucose + high-homocysteine conditions (Figure 1, Figure 2 and Figure 3).

### 3.2. In Vivo Mouse Model

**Figure 4:**
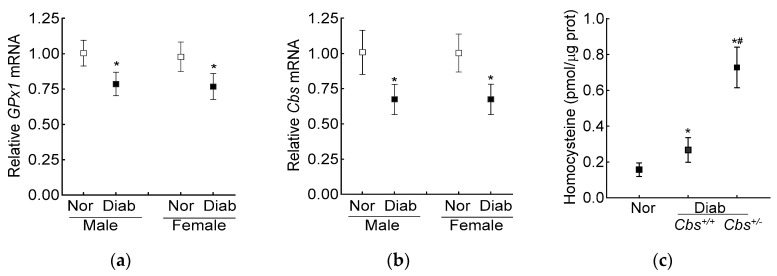
Effect of mouse gender on retinal *GPx1* and *Cbs* gene transcripts. (**a**) *GPx1* and (**b**) *Cbs* gene transcripts were quantified in the retina from *Cbs*^+/+^ mice diabetic for four months using *18S rRNA* as the housekeeping gene. (**c**) Total retinal homocysteine levels were quantified by an ELISA method. Each measurement was performed in duplicate with 5–6 mice/group, and the results are presented as mean ± SD. Nor = normal and Diab = diabetes. *Cbs*^+/+^ and *Cbs*^+/−^ = *Cbs* wildtype and heterozygous mice, respectively. * *p* < 0.05 vs normal and ^#^ *p* < 0.05 vs. *Cbs*^+/+^ diabetic mice.

The average blood glucose in the male and female diabetic mice (*Cbs^+/−^* or *Cbs^+/+^)* was similar (~350 mg/dL) throughout the duration of the experiment. Compared to the age- and sex-matched nondiabetic mice, the decrease in retinal *Cbs* and *GPx1* gene transcripts in the mice diabetic for four months was comparable in both the genders (Figure 4a,b). Furthermore, our previous studies have shown a similar retinal histopathology and mitochondrial dysfunction in male and female mice [40], supporting the idea that the severity of hyperglycemia and the development of diabetic retinopathy were not influenced by the gender of the mouse; the following results were pooled from both genders of mice. 

The increase in homocysteine levels, as expected [23], was ~two- and over seven-fold higher in the *Cbs^+/+^* and *Cbs^+/−^* diabetic mice, respectively, compared to the nondiabetic mice, confirming severe hyperhomocysteinemia (Figure 4c). 

**Figure 5:**
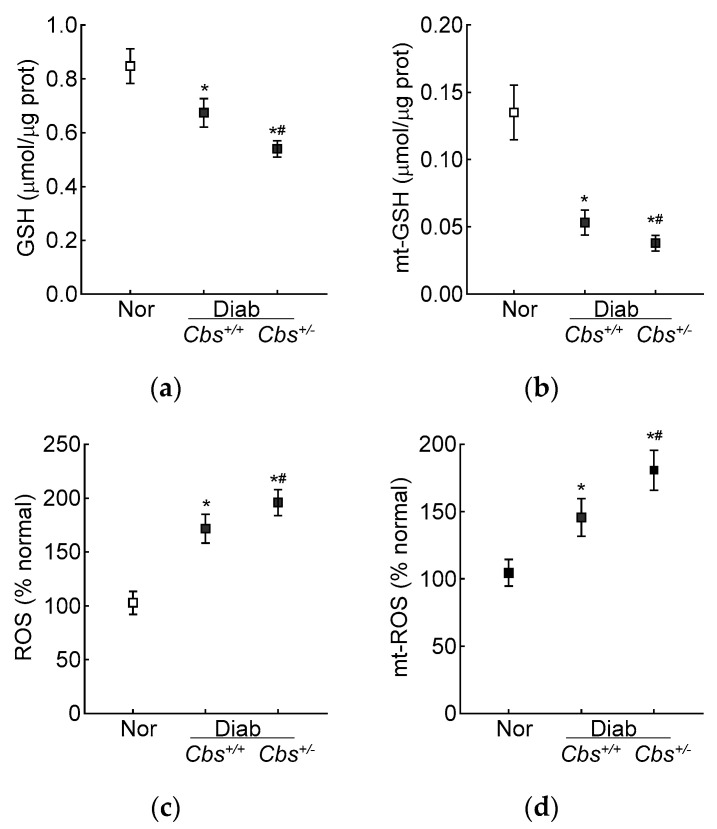
Retinal GSH–GPx in hyperglycemia–hyperhomocysteinemic mouse model. (**a**) Total and (**b**) mitochondrial GSH levels were quantified by an enzymatic recycling method by quantifying 2-nitro-5-thiobenzoate, and (**c**) total (by DCFH-DA) and (**d**) mitochondrial (by MitoSox red) ROS levels were quantified by fluorometric method. Measurements were made in duplicate with 5–6 mice/group. The data are presented as mean ± SD. * *p* < 0.05 vs. normal and ^#^ *p* < 0.05 vs. *Cbs*^+/+^ diabetic mice.

Compared to the normal mice, the retinal total GSH levels were decreased by 30% and ~50% and ROS levels were increased by ~70% and >2-fold in the *Cbs*^+/+^ diabetic mice and *Cbs*^+/−^ diabetic mice, respectively. In the same *Cbs*^+/+^ and *Cbs*^+*/−*^ diabetic mice, the mt-GSH levels were also decreased and mt-ROS levels were elevated by 50–70% compared to age-matched nondiabetic mice. The values in the *Cbs*^+/−^ diabetic mice were significantly different from those in the *Cbs^+/+^* diabetic mice (*p* < 0.05) (Figure 5a–d). 

**Figure 6:**
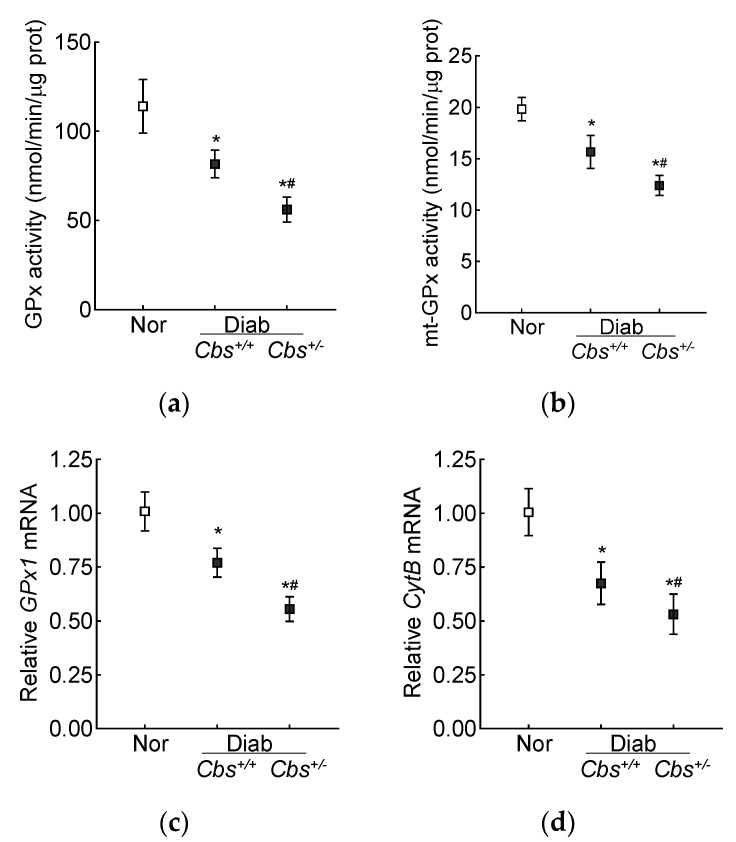
Effect of hyperglycemia + hyperhomocysteinemia on retinal GPx. (**a**) Total and (**b**) mitochondrial GPx activity was measured in 15 µg of protein. Gene transcripts of (**c**) *GPx1* and (**d**) *CytB* were quantified by qRT PCR, and *18S rRNA* was used as a housekeeping gene. The results are mean ± SD obtained from 6–7 mice/group, with each measurement made in duplicate. Nor = normal; *Cbs*^+/+^/Diab and *Cbs*^+/−^/Diab = *Cbs* wildtype and heterozygous mice, diabetic for four months. * *p* < 0.05 vs. normal and ^#^ *p* < 0.05 vs. *Cbs*^+/+^ diabetic mice.

Retinal GPx activity was inhibited by 30% in the *Cbs*^+/+^ diabetic mice and by ~50% in the *Cbs*^+/−^ diabetic mice compared to nondiabetic mice, and the activity of mt-GPx was inhibited by 50–70% (Figure 6a,b). Gene transcripts of *GPx1* were also decreased by 30% in the *Cbs*^+/+^ diabetic mice and by 50% in the *Cbs*^+/−^ diabetic mice compared to their age-matched normal mice (Figure 6c). Consistent with the decrease in mt-GSH and mt-GPx and the increase in mt-ROS, gene transcripts of mtDNA-encoded *CytB* were also decreased by 30–50% in the *Cbs*^+/+^ and *Cbs*^+/−^ diabetic mice, confirming mitochondrial damage (Figure 6d). 

**Figure 7:**
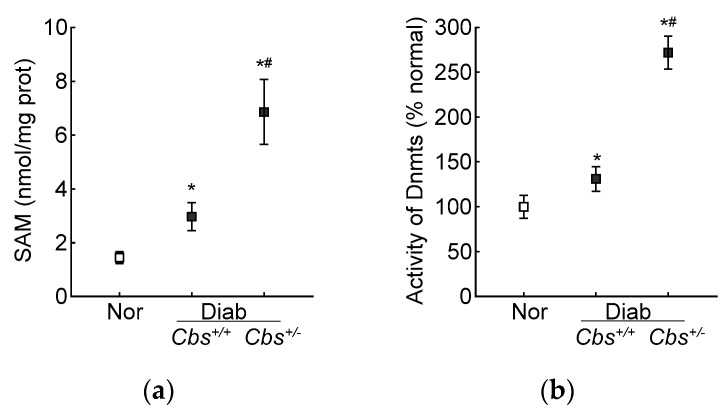

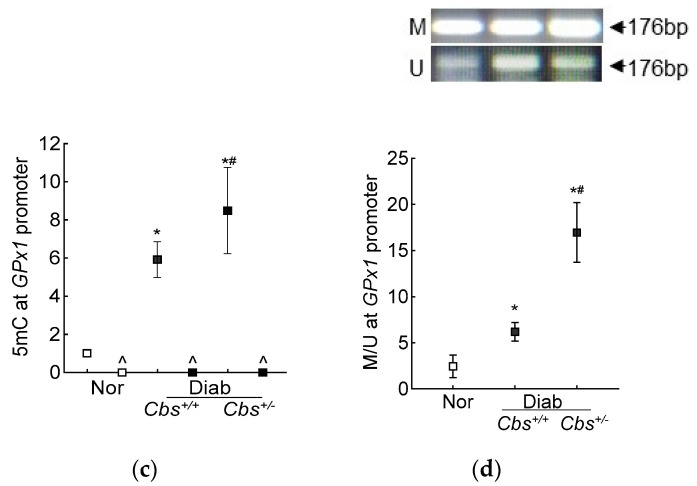
Retinal *Gpx1* promoter DNA methylation. Retinal (**a**) SAM levels were quantified by ELISA, (**b**) activity of Dnmts by DNA Methyltransferase Activity assay kit. (**c**) 5mC levels at the *Gpx1* promoter DNA were measured by methylated DNA immunoprecipitation method. (**d**) Methylation-specific PCR was performed using bisulfite-converted DNA. Each measurement was performed in duplicate in 5–6 mice/group, and the results are presented as mean ± SD. The values obtained from normal mice were considered as 1 or 100%. Nor = normal *Cbs*^+/+^ mice; *Cbs*^+/+^/Diab and *Cbs*^+/−^/Diab = *Cbs* wildtype and heterozygous mice, diabetic for four months; M = methylated; U = unmethylated. ^ = IgG control; * p < 0.05 vs. normal and ^#^ *p* < 0.05 vs. *Cbs^+/+^* diabetic mice.

In accordance with the results from the HRECs, in the *Cbs*^+/+^ diabetic mice, the retinal SAM levels and Dnmts activity were elevated by three-fold and 50%, respectively, and Dnmts activation was significantly higher in the hyperhomocystinemic–hyperglycemia model, with an over six-fold increase in SAM and >250% increase in Dnmts activity in the retina from the *Cbs*^+/−^ diabetic mice (Figure 7a,b). Consistent with the increase in SAM and Dnmts, *GPx1* promoter DNA methylation was also increased significantly in both the *Cbs*^+/+^ and *Cbs*^+/−^ diabetic mice compared to normal mice; the 5mC levels at the *GPx1* promoter DNA were five- and seven-fold higher in the *Cbs*^+/+^ and *Cbs*^+/−^ diabetic mice, respectively (Figure 7c), and they were also significantly different from each other (*p* < 0.05). The ratio of methylated *GPx1* promoter DNA to unmethylated *GPx1* promoter DNA was significantly higher in the *Cbs*^+/+^ diabetic mice, compared to normal mice (Figure 7d), and was further increased significantly in the *Cbs*^+/−^ diabetic mice (*p* < 0.05 vs. *Cbs*^+/+^).

## 4. Discussion

Diabetic retinopathy is a multi-factorial disease, and although hyperglycemia is the main initiator, the pathogenesis of this blinding disease is influenced by many systemic factors, including hyperlipidemia and hypertension [15,41,42]. Hyperhomocysteinemia is another problem faced by many diabetic patients and is shown to have a positive correlation with the progression of diabetic retinopathy [16,43]. Experimental models have documented its role in mitochondrial dysfunction and have shown that hyperhomocysteinemia, in a hyperglycemic milieu, accelerates and exacerbates mitochondrial dysfunction and fragmentation and impairs the removal of damaged mitochondria [20,42,44]. Here, our results demonstrated that hyperhomocysteinemia in a hyperglycemic environment further decreases intracellular antioxidant GSH levels and the antioxidant defense enzyme GPx, which uses GSH as a cofactor. Compared to the total cellular levels, the antioxidant defense system in mitochondria is further downregulated with lower mt-GSH levels and mt-GPx activity and higher mt-ROS. We have also elucidated a mechanism for the downregulation of GPx; the data demonstrate that *GPx1* promoter DNA is hypermethylated in a hyperglycemic medium, and homocysteine, by elevating SAM levels, enhances the activation of DNA methylation machinery, increasing promoter methylation. This further downregulates *GPx1* gene transcripts and augments mt-ROS accumulation and mitochondrial damage. Thus, this study further strengthens the importance of maintaining normal homocysteine levels for diabetic patients to prevent/retard the development of diabetic retinopathy.

Glutathione is the main nonprotein thiol found in cells and is the most abundant antioxidant molecule in living organisms [45]. It helps to maintain the intracellular redox milieu to preserve the thiol–disulfide redox states of proteins, and it is also involved in cellular signaling and the redox activation of transcription factors. Intracellular glutathione homeostasis involves a fine balance between its synthesis, consumption and degradation [46]. Although GSH is biosynthesized in the cytosol, it is also distributed in other intracellular organelles, including mitochondria and the nucleus [12]. Mitochondria have a minor fraction of the total GSH pool (10–15%), but the GSH concentration in mitochondria is similar to that of cytosol (10–14 mM) [47]. Within the mitochondrial matrix, hydrogen peroxide is produced by the conversion of O_2_^•−^ by manganese superoxide dismutase, but mitochondria lack catalase, and GSH in mitochondria is the only defense available to detoxify hydrogen peroxide [48]. Here, our results showed that, consistent with the decrease in total GSH levels and GPx activity in the hyperglycemic milieu, GSH levels and GPx activity are also decreased in mitochondria. Homocysteine supplementation, in addition to decreasing total GSH levels and GPx activity, further decreases mt-GSH levels and mt-GPx activity, thereby increasing mt-ROS accumulation. In support, homocysteine metabolism, via a trans-sulfuration process, forms a sulfur metabolite, cystathionine, and the hydrolysis of cystathionine by cystathionine γ-lyase forms α-ketobutyrate and cysteine, and cysteine is one of the three amino acids required for GSH biosynthesis [49]. High homocysteine levels are associated with reduced activities of the electron transport chain and increased oxidative stress [44,50], and homocysteine-mediated endothelial dysfunction, in part, is associated with a reduction in GPx1, which is ameliorated by *GPx1* overexpression [51]. Furthermore, in the hyperglycemic milieu, mitochondrial damage and the development of diabetic retinopathy is accelerated and excerbated [19,20].

Our results show that, in addition to the reduction in GPx activity, gene transcripts of *GPx1* are also decreased in hyperglycemia, and additional homocysteine further reduces *GPx1* gene transcripts. Among the eight members of the GPx family of enzymes, GPx1 is the major isoform that reduces hydrogen peroxide to water using GSH as a cofactor. Although it is a cytosolic enzyme, a small fraction of GPx1 is also present in mitochondria [50]. In diabetic retinopathy, DNA methylation machinery is activated, and hypermethylation of several genes is implicated in the impaired mitochondrial homeostasis [4]. The promoter region of *GPx1* has GC-boxes, and its aberrant hypermethylation is seen in chronic diseases [26]. Here, we showed that retinal *GPx1* promoter DNA is hypermethylated in diabetes and that SAM levels are elevated. The hypermethylation of *GPx1* promoter, the activation of Dnmts and the increase in SAM levels are exacerbated by the supplementation of hyperglycemic insult with homocysteine. Homocysteine is shown to disrupt the balance between SAM and S-adenosylhomocysteine, increasing SAM levels, and increased levels of this major methyl donor activate DNA methylation [52]. Consistent with the results presented here, homocysteine suppresses the transcription of a tissue inhibitor of matrixmetalloproteinase-9, *Timp1*, by hypermethylating DNA at its promoter, increasing mitochondrial damage, and it also accelerates the development of diabetic retinopathy [20,28].

## 5. Conclusions

Using experimental models of diabetic retinopathy, the present study demonstrates that hyperhomocysteinemia, in a diabetic environment, decreases mitochondrial GSH–GPx levels, thus increasing mitochondrial ROS and damage. In addition to the functional inhibition of GPx, this study also clearly shows the role of homocysteine in *GPx1* promoter DNA methylation, which further suppresses its transcription. The decrease in GPx–GSH in mitochondria augments their damage, and the damaged mitochondria continue to self-propagate the vicious cycle of free radicals. Thus, this study strengthens the importance of regulating homocysteine levels in diabetic patients to impede the development/progression of the blinding disease.

## Figures and Tables

**Table 1:** Primer sequence.

Primer	Sequence (5′-3′)	Accession No.	Amplicon (bp)
Human			
*GPx1*	Fwd-CAACCAGTTTGGGCATCAGG Rev-AGAGAGTAGCCAGACTCTCCG	NM_201397.3	134
*Cbs*	Fwd-GACCAAGTTCCTGAGCGACA Rev-CGGAGGATCTCGATGGTGTG	NM_000071.3	171
*Mat1a*	Fwd-GCGTGAGTGGAGAAGTGTGA Rev-CCAGTCAGTCCAAGACAGCC	NM_000429.3	142
*CytB*	Fwd-TCACCAGACGCCTCAACCGC Rev-GCCTCGCCCGATGTGTAGGA	ENSG00000198727	138
*B-actin*	Fwd-AGCCTCGCCTTTGCCGATCCG Rev-TCTCTTGCTCTGGGCCTCGTCG	NM_001101.5	237
*GPx1* Promoter (MeDIP)	Fwd-GGGGGCCGGATGAGGCGGGA Rev-AACTGGCCGGCGGCGGGTCAC	NM_201397	71
*GPx1* Promoter (MS-PCR)	Methyl Fwd-GTTTTTTTCGGTTTAGGAGGAGTAC Rev-CGTAACACAAACAATTTTCCGA	NM_201397	144
	Unmethyl Fwd-TTTTTTTTGGTTTAGGAGTATG Rev-TCACATAACACAAACAATTTTCCA		148
Mouse			
*GPx1*	Fwd-GACACCAGGTATATGGGGCG Rev-AGAAAGTAAGCGGTGTCCCG	NM_008160.6	149
*Cbs*	Fwd-CGTGCTCTCCATCCTAGCAC Rev-GTTGGCTCTTGAGTCCCCTC	NM_144855.4	137
*Mat1a*	Fwd-GCAGAAGTCATCTCCTCGGG Rev-ACAGGTCCATTCATTGTGCCA	NM_133653.3	146
*CytB*	Fwd-ACCCGCCCCATCCAACATCTCAT Rev-TTGAGGCTCCGTTTGCGTGT	AB819918.1	203
*18S rRNA*	Fwd-GCCCTGTAATTGGAATGAGTCCACTT Rev-CTCCCCAAGATCCAACTACGAGCTTT	NR_003278.3	148
*GPx1* Promoter (MeDIP)	Fwd-AACATCTCCAGTATGTGTG Rev-GACATTCTCAATGAGCAG	NM_008160	141
*GPx1* Promoter (MS-PCR)	Methyl Fwd-GTAGGGTTTTGTGAGCGTTAGTAC Rev-AATAAACAACACCTTACCCCG	NM_008160	176
	Unmethyl Fwd-TAGGGTTTTGTGAGTGTTAGTATGG Rev-CAATAAACAACACCTTACCCCAC		176

## Data Availability

R.A.K. is the guarantor of this work and, as such, has full access to all data in the study and takes responsibility for the integrity of the data and the accuracy of the data analysis.
